# Improved Insulating Properties of Polymer Dielectric by Constructing Interfacial Composite Coatings

**DOI:** 10.3390/ma17010059

**Published:** 2023-12-22

**Authors:** Jia-Xuan Wang, Yong-Gang Chen, Ji-Ming Chen, Zhi-Hui Yin, Chun-Song Chen, Yi-Fei Li, Ting Deng, Xiao-Bo Guo, Ming-Xiao Zhu

**Affiliations:** 1 College of New Energy, China University of Petroleum (East China), Qingdao 266580, China; 2 School of Science, China University of Petroleum (East China), Qingdao 266580, China; chenyg@upc.edu.cn

**Keywords:** breakdown strength, conduction current, trap distribution, surface coating, spraying

## Abstract

Polymeric dielectrics exhibit remarkable dielectric characteristics and wide applicability, rendering them extensively employed within the domain of electrical insulation. Nevertheless, the electrical strength has always been a bottleneck, preventing its further utilization. Nanocomposite materials can effectively improve insulation strength, but uniform doping of nanofillers in engineering applications is a challenge. Consequently, a nanocomposite interfacial coating was meticulously designed to interpose between the electrode and the polymer, which can significantly improve DC breakdown performance. Subsequently, the effects of filler concentration and coating duration on DC breakdown performance, high field conductivity, and trap distribution characteristics were analyzed. The results indicate that the composite coating introduces deep traps between the electrode-polymer interface, which enhances the carrier confinement, resulting in reduced conductivity and enhanced DC breakdown strength. The incorporation of a composite coating at the interface between the electrode and polymer presents novel avenues for enhancing the dielectric insulation of polymers.

## 1. Introduction

Polymers, such as polyethylene and polypropylene, have been widely used in various power equipment, including cables and capacitors, due to their remarkable insulation, mechanical, and chemical performances [1,2], and they have attracted scholars to study their related properties [3,4]. However, during the long-term operation of cables, various defects can generate distorted electric fields, accelerating insulation aging and even causing premature insulation failure. This significantly impacts the lifespan of insulation [5,6,7]. Therefore, there is an urgent need to elevate the insulation performance of polymer dielectrics. 

The insulation performance of polymer dielectrics can be substantially enhanced through the incorporation of nanofillers. The effect of improvement is intricately related to the properties of the nanoparticles, such as their composition, doping concentration, particle dimensions, morphology, and surface characteristics [8,9,10,11]. Typical nanofillers include wide-band gap inorganic fillers (MgO [12,13], Al_2_O_3_ [14,15], SiO_2_ [16,17], etc.) and conductive fillers (graphene [18,19,20], carbon black [21,22,23], etc.). Numerous scholars have analyzed the electrical insulation properties of polymers when doped with nanoparticles and found that the space charge accumulation behavior and breakdown strength can be effectively improved. The main reason is that the incorporation of nanoparticles introduces deep traps between the interface of nanofiller and matrix, which is conducive to the accumulation of homopolar charge at the interface of electrode and polymer, and the resulting reverse electric field inhibits the further injection of electrode charge and suppresses the accumulation of space charge [24,25]. 

Nevertheless, nanoparticles typically possess high surface energy, which results in the agglomeration of nanoparticles within the polymer matrix, thereby impeding the improvement of the insulation performance of the polymer material [26,27]. Considering that most space charge accumulation takes place at the interface between electrodes and polymers, it is therefore more effective to alleviate space charge accumulation by manipulating the trap distribution at the interface rather than doping nanoparticles into the polymer matrix. Currently, the prevailing techniques employed for interface tailoring encompass primarily surface fluorination treatment [28,29], magnetron sputtering [30,31], plasma surface modification [32,33], and chemical vapor deposition [34,35,36], among others. However, it is noteworthy that these methods typically necessitate the utilization of intricate experimental apparatus. 

Nanocoating is a flexible method to regulate interface trap distribution characteristics through tailoring the physical properties, morphology, and contents of fillers. Therefore, it is particularly suitable for improving insulation performance by adjusting the trap characteristics of nanocoating. Meng et al. effectively suppressed the accumulation of space charge inside the XLPE by spraying a trace amount of EPDM@ZnO solution at the interface [37]. Wang et al. sprayed a zinc oxide/polydimethylsiloxane (ZnO/PDMS) coating on the surface of PTFE and found that the breakdown strength and the flashover voltage were significantly improved [38]. In conclusion, composite coatings at the interface exhibit a remarkable capability to enhance the dielectric insulation performance of polymers. However, it should be noted that research in this field remains relatively limited, and further exploration of the modification mechanisms of interface coatings is significant. 

In this paper, composite coatings with carbon quantum dots (CQDs) are presented to improve the insulation performance of low-density polyethylene (LDPE). Varied composite coatings with different trap states are synthesized by adjusting the concentration of the filler and the duration of the spraying process, and the direct current breakdown characteristics, high field conductivity, and trap distribution properties are investigated. The findings manifest that the interface composite coating effectively enhances the insulation efficacy of the LDPE film, which provides a novel route for improving insulation properties. 

## 2. Experimental Section

### 2.1. Materials

The LDPE pellets utilized were supplied by Dow Chemical Company (Midland, MI, USA), with a density of 0.92 g/cm^3^ and a melt index (190 °C/2.16 kg) of 0.2 g/10 min. The material had a number-average molecular weight (Mn) of 30,129 g/mol and a weight-average molecular weight (Mw) of 89,524 g/mol. Xylene (purity 99%) was purchased from Shanghai Aladdin Biochemical Technology Co., Ltd. (Shanghai, China). Dithiobisbenzoic acid (DTSA, 98%) was purchased from Shanghai Marklin Biochemical Co., Ltd. (Shanghai, China), and acetic acid (99.5% purity) was purchased from Shanghai Myriad Chemical Co., Ltd. (Shanghai, China), without further purification.

### 2.2. Sample Preparation

LDPE Film Preparation: Pure polyethylene films were prepared by hot pressing LDPE pellets. Firstly, the polyethylene samples were preheated at 120 °C for 5 min and then pressed at 180 °C for 15 min at a pressure of 10 MPa to produce the polyethylene samples. Next, the prepared samples were placed in a vacuum drying oven and degassed at 70 °C for 12 h to eliminate cross-linking by-products. Finally, the sample surface was polished by 1000-grit mesh alumina sandpapers for about 5 min to make the surface smoother and flatter.

CQDs Preparation: CQDs were prepared by the solution chemical method [39]. 490 mg of DTSA powder was dissolved in 40 mL of acetic acid solution and stirred until the DTSA was completely dissolved. The mixed solution was then transferred to a Teflon reactor with a volume of 100 mL and placed in a vacuum oven at 180 °C for 10 h. The cooled reagents were transferred to a beaker containing 500 mL of boiling water and stirred in an oil bath at 105 °C for 1 h to volatilize the unreacted acetic acid solvent. After that, the solution containing CQDs was filtered, and the solid material obtained was dried at room temperature as pure CQDs powder.

**Figure 1:**
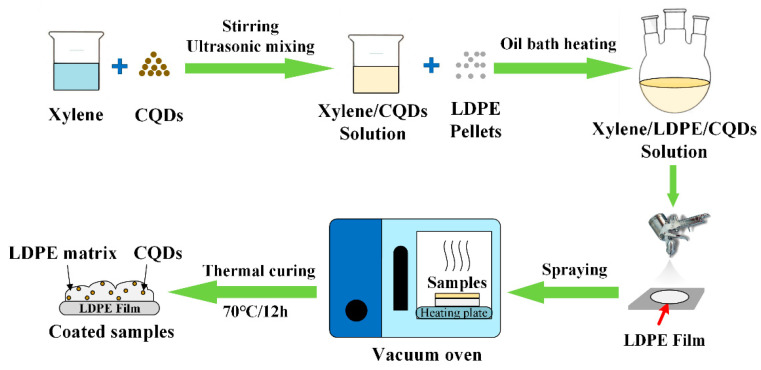
The schematic diagram for preparation of the electrode–polymer interface composite coatings.

Coating Preparation: The schematic diagram of composite coating preparation is shown in Figure 1. Polyethylene was selected as the matrix and CQDs as fillers for the preparation of composite coatings. The desired mass fraction of CQD powder was added to 50 mL of xylene solvent, stirred, and ultrasonically dispersed for 1 h to form a mixed solution. Afterwards, the mixed solution was poured into a round-bottomed flask, and 1 g of polyethylene particles was added and dissolved by magnetic stirring at 120 °C until completely mixed. The solution was then sprayed onto the polyethylene film, carefully controlling the gun pressure (45 psi), spraying distance (200 mm), and spraying time. Finally, the sprayed samples were cured in an oven at 70 °C for 12 h. The mass fractions of nanofiller to polymer matrix were 0.1 wt%, 0.2 wt%, 0.3 wt%, 0.5 wt% and 0.7 wt%, respectively. The spraying time was also adjusted to 4 s, 6 s, 8 s, 12 s, and 16 s. The different coatings were expressed as “CQDs-mass fraction-spraying time”. For example, CQDs-0.3-8 s is a coating filled with 0.3 wt% CQDs filler sprayed for 8 s.

### 2.3. Material Characterization and Electrical Properties

Material characterization: The surface morphologies of the coatings and elemental distributions were determined using a scanning electron microscope (MIRA LMS, Tescan, Brno, Czech Republic). The thickness of the samples was measured with a thickness gauge. 

DC Breakdown Strength Test: The DC breakdown strength test was performed by placing the samples with an average thickness of 50 μm between two copper spherical electrodes with a diameter of 13 mm, and the computer-controlled voltage was applied at a ramp rate of 1 kV/s until the specimen broke down, with 15 sets of measurements for each type of specimen. The DC breakdown strength was analyzed within the framework of a two-parameter Weibull statistic described as:

(1) $$P\left(E_{s}\right)=1-\exp\left(-{\left(E_{s}/\alpha \right)}^{\beta }\right)$$

where $P\left(E_{s}\right)$ is the cumulative probability of DC breakdown, $E_{s}$ is the measured DC breakdown strength, the scale parameter α is the strength for which there is a 63.2% probability for the samples to breakdown (Weibull breakdown strength), the shape distribution parameters β evaluate the scatter of the measured data, and a higher β means greater dielectric reliability.

DC electrical conductivity test: A three-electrode system with a temperature-controlled oven and a high-precision ammeter were used to measure the current density of the specimens with a thickness of 200 μm under different electric field strengths. The test sample was placed in a short-circuit in a vacuum drying box for 12 h before the test at a temperature of 60 °C. 

Trap distribution characteristics: The trap distribution characteristics of the composite coatings were measured by the isothermal surface potential decay (ISPD) method. A specimen with a thickness of 200 μm was selected as the sample to be tested by ISPD, and the surface of the specimen was charged by the corona discharge method. A “needle grid-to-surface” electrode structure was established to deposit charges on the sample surface, and DC voltage of ±6 kV and ±2 kV was applied to the needle electrode and the metal mesh, respectively, for 5 min. The charged specimens were transferred to a Kelvin probe (Trek 347, Advanced Energy, Denver, CO, USA) via a displacement stage to measure the transient surface potential. The experimental specimens were heated using a hot plate, and the temperature was maintained at 50 °C throughout the measurement. According to the theory of isothermal discharge current (IDC) proposed by Simmons, the trap level and density of traps can be determined with the following equations:

(2) $$E_{T}=kT\ln\left(\gamma t\right)$$

(3) $$N\left(E_{T}\right)=\frac{\varepsilon t}{q_{e}f_{0}kT\delta L}\frac{d\varphi \left(t\right)}{dt}$$

where $E_{T}$ is the trap level. k is the Boltzmann constant. T is the absolute temperature (323 K). γ is the escape frequency of trapped hole/electrons, which is approximately equal to 10^12^ s^−1^. t is the measurement time in s. $N\left(E_{T}\right)$ is the density of traps. ε and L are the permittivity and thickness of the specimen, respectively. $q_{e}$ is the elementary charge quantity. $f_{0}$ is the rate of initial occupancy of traps, which is assumed to be 1 here. δ is the penetration depth of injected charges, which is set as 2 μm. φ(t) is the potential measured with the Kelvin probe.

During the isothermal potential decay process, charges in shallower traps detrap easier than those in deeper traps. That is, the release time of charges is dependent on its trap depth. As a result, two kinds of decay processes occur simultaneously. Therefore, the surface potential decay curve can be fitted by the double exponential equation:

(4) $$\varphi \left(t\right)=a_{1}e^{-b_{1}t}+a_{2}e^{-b_{2}t}$$

where $a_{1}$, $a_{2}$, $b_{1}$, and $b_{2}$ are the fitting parameters. $b_{1}$, and $b_{2}$ represent the decay time constant for deep and shallow traps, respectively. Then the trap distribution can be obtained by substituting the fitting results into the equation.

## 3. Results and Discussion

### 3.1. Characterization

**Figure 2:**
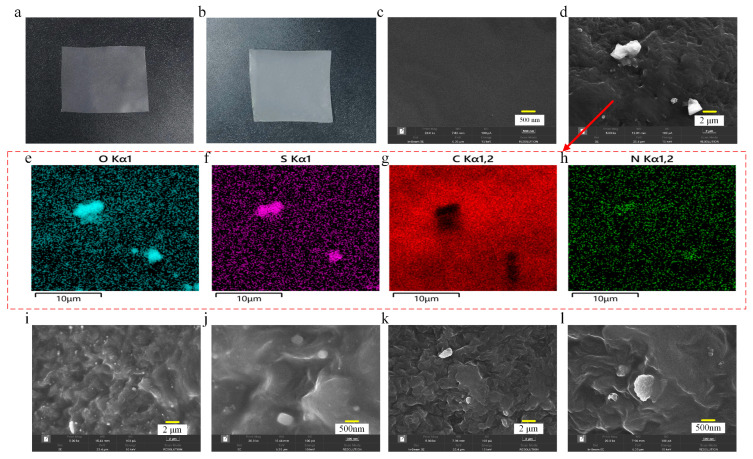
Characterizations of LDPE before and after coating. Photographs of LDPE (**a**) and coated LDPE (**b**). SEM images of uncoated LDPE (**c**) and coated LDPE (**d**). EDS mapping images of elements on coated LDPE (**d**) with O (**e**), S (**f**), C (**g**) and N (**h**). SEM images of composite coating imbued with a 0.3 wt% CQD filler with a magnification of 5000 (**i**) and 20,000 (**j**). SEM images of composite coating imbued with a 0.2 wt% CQD filler with a magnification of 5000 (**k**) and 20,000 (**l**).

Figure 2a,b shows the photographs of two kinds of samples. The composite coating is observed to possess a white coloration, intermingled with a faint manifestation of yellowish-brown tones. Figure 2c–l depicts the scanning electron microscope (SEM) results, which illustrate the surface morphology of the pristine polyethylene film and coated sample. It can be found that the surface of pristine polyethylene is relatively smooth. Instead, tiny white particles are found on the surface of the coated sample, which are embedded in the polyethylene matrix and dispersed in an island-like manner, and the polyethylene matrix is undulated in the shape of hills. In addition, the chemical characteristics of the coated sample are further examined through energy dispersive spectrometer (EDS) analysis of O, S, C, and N to confirm whether the white particles are CQDs. Figure 2e–h shows that additional O, S, and C are found on the surface of the coated sample, which are mainly concentrated in the white particles, demonstrating that these white particles are the CQDs in the coating. Figure 2i–l demonstrates the difference in the surface morphology of the coatings at CQD doping concentrations of 0.3 wt% and 0.2 wt%. It can be clearly seen that the CQD particles in the coating also increase significantly when the doping concentration is higher.

### 3.2. DC Breakdown Strength

**Figure 3:**
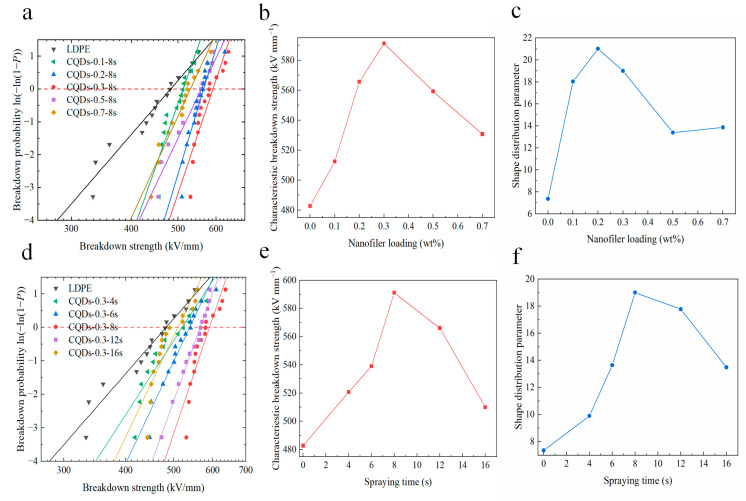
DC breakdown characteristics of different coated samples. Weibull distribution plot of DC breakdown strength (**a**), characteristic breakdown strength (**b**), and shape distribution parameters (**c**) of coated samples doped with varying concentrations of CQDs; Weibull distribution plot of DC breakdown strength (**d**), characteristic breakdown strength (**e**), and shape distribution parameters (**f**) of coated samples prepared with varying spraying durations.

The DC breakdown characteristics of the pristine LDPE and coated samples are illustrated in Figure 3, in which the DC breakdown results are analyzed utilizing the double-parameter Weibull distribution. Figure 3a–c depicts the DC breakdown strength of composite coatings doped with varying concentrations of CQDs. The results indicate that the breakdown strength of polyethylene is significantly improved by the application of coatings. And the degree of improvement varies with the concentration of CQDs. Notably, the DC breakdown strength first increases, then decreases with the concentration of CQDs, reaching its peak increment at a concentration of 0.3 wt%. As can be seen from the scale parameter in Figure 3b, the breakdown strength of the pristine LDPE is 482.785 kV/mm. The application of the CQD composite coating yields a profound enhancement in the DC breakdown strength. When the doping concentration attains 0.3 wt%, the sample exhibits a maximum breakdown strength of 591.109 kV/mm, surpassing that of pure LDPE by 22.4%. As depicted in Figure 3c, the shape parameters β exhibit alterations in response to varying doping concentrations of CQDs. Notably, the shape parameters of coated samples surpass those of pure LDPE, suggesting that the incorporation of composite coatings engenders a diminishment in the dispersion of the breakdown strength, resulting in a more stable insulation performance.

Figure 3d–f depicts the Weibull distribution analysis of the DC breakdown strength of LDPE and composite coatings with varying spraying durations. Evidently, the DC breakdown strength of the composite coating surpasses that of pure LDPE. Furthermore, it exhibits an initial ascending trend followed by a subsequent decline, with the most substantial enhancement observed at an 8-second spraying duration. Figure 3e elucidates the marked augmentation in the DC breakdown strength of composite coating samples. Specifically, the DC breakdown strength exhibited an approximate escalation of 7.8%, 11.6%, 22.4%, 17.2%, and 5.6% with a spraying duration of 4, 6, 8, 12, and 16 s, respectively. Furthermore, Figure 3f illustrates the variation of shape distribution parameters, denoted by β, in response to changes in spraying time. Notably, the introduction of the coating results in a substantial increase in the β value, which significantly mitigates the dispersion of breakdown strength. It is hypothesized that deep traps generated in the coating film may be the main reason for improving the breakdown strength, which will be discussed in the following sections.

### 3.3. High Field Conductance Characteristics

**Figure 4:**
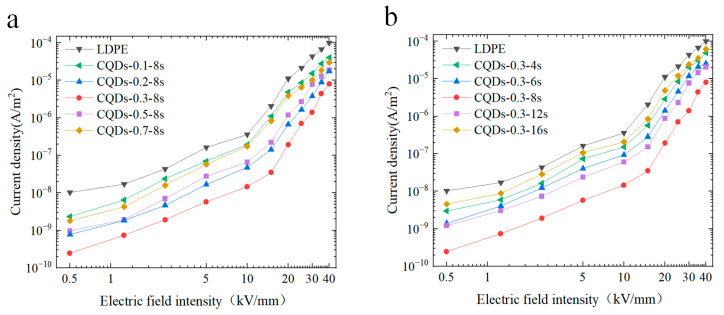
(**a**) High-field conductivity curve between electric field and current density of coated samples doped with varying concentrations of CQDs; (**b**) high-field conductivity curve between electric field and current density of coated samples prepared with varying spraying durations.

Figure 4 compares the electrical conductivity of the coated samples with pristine LDPE. Specifically, the current density—electric field (*J–E*) characteristic curves of LDPE and composite coatings, doped with varying concentrations of CQDs, are represented in Figure 4a. As illustrated in Figure 4a, a pronounced alteration in the slope of the *J–E* curves is observed with the increase of the electric field. This behavior aligns with the phenomenon associated with the space charge-limiting current exhibited by solid dielectrics [40]. For LDPE, the conductivity current reveals two distinct gradients with a turning point of 10 kV/mm. Specifically, the *J–E* curve exhibits a relatively modest slope when the field strength lies below 10 kV/mm, indicating a small alteration in current density in response to the field strength. However, at 10 kV/mm, a pivotal transition in the conductivity current occurs, whereby the curve assumes a steeper slope. The conductivity current of the coated samples exhibits a lower magnitude in comparison to that of LDPE, displaying a trend of initial decline followed by subsequent escalation in response to the CQD doping concentration. Remarkably, the coated samples exhibit a turning electric field in the range of 10–15 kV/mm, which consistently exceed the turning field strength observed in LDPE. Notably, at a doping concentration of 0.3 wt%, the current density attains its minimum value among all specimens, and the turning field strength reaches approximately 15 kV/mm, surpassing the corresponding value of LDPE. In line with the theory of space charge-limiting current, the magnitude of the transitional electric field corresponds to the alteration in the conductivity mechanism. When the electric field is above the transition point, the formation of space charge begins to limit the change in current. 

A parallel trend is also manifested in the *J–E* curves of coated samples synthesized at varying spraying durations, as illustrated in Figure 4b. The conductivity current of the composite coating exhibits a lower magnitude compared to LDPE, particularly for the coated sample prepared through an 8 s spraying duration. With an increase in spraying time, the conductivity current of the composite coating initially diminishes, only to subsequently augment. Notably, the composite coating sample achieved through an 8 s spraying interval attains the minimum conductivity current and the highest turning electric field.

The pertinent investigation concerning the turning field intensity suggests its correlation with the profundity of traps within the entire sample. The turning electric field can be employed to ascertain the depth of traps within the sample [41]. In summary, the reason for the decrease in conductivity of the composite coating is closely related to its trap distribution. In comparison to pristine LDPE, the integration of composite coatings introduces a substantial abundance of deep traps at the electrode–polymer interface (see Section 3.4). These deep traps effectively impede the migration of carriers injected from the electrode [42]. In comparison to LDPE, the coated samples exhibit a markedly heightened trap depth, with the coated sample imbued with a 0.3 wt% CQDs filler demonstrating the utmost depth of traps. Consequently, this manifests as a reduction in carrier concentration and mobility, thereby leading to a considerable diminution in conductivity current compared to that of LDPE. When the CQDs exceed a certain threshold or spraying nanoparticles with a considerable coating density, the interfacial regions of nanoparticles overlap, leading to a reduction in the depth of traps present in the coating, which results in an increase in the conductive current of the material.

### 3.4. Trap Distribution Characteristics

**Figure 5:**
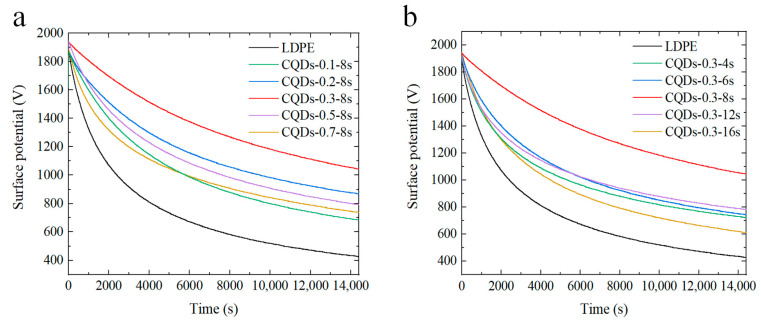
(**a**) Isothermal surface potential decay characteristics of coated samples doped with varying concentrations of CQDs; (**b**) isothermal surface potential decay characteristics of coated samples prepared with varying spraying durations.

It is widely accepted that charge traps can significantly affect charge migration behaviors and thus further affect breakdown strength. In order to gain insight into the improving mechanism of composite coatings on insulation performances, the surface potential decay curves and trap distribution were measured. The surface potential decay curves of composite coatings with different concentrations of CQDs and spraying durations were analyzed, as shown in Figure 5a,b. It can be found that the surface potential decay curve of the composite coatings with CQD concentrations in the range of 0.1–0.3 wt% is flatter than the pure LDPE sample. Furthermore, the decay rate decreases as the mass fraction increases. As the concentration of CQDs increases from 0.3 wt% to 0.7 wt%, a notable acceleration in the surface potential decay rate of the composite coating is observed.

Analogously, as depicted in Figure 5b, it is evident that the potential decay rate of composite coatings manufactured with different spraying durations is significantly reduced in comparison to pure LDPE. It is worth noting that the inhibition of the charge transfer process is most significant when the spraying duration exceeds 8 s. This finding suggests that the incorporation of the electrode polymer interface composite coating effectively mitigates the infiltration of surface charges into the inner regions of the sample.

**Figure 6:**
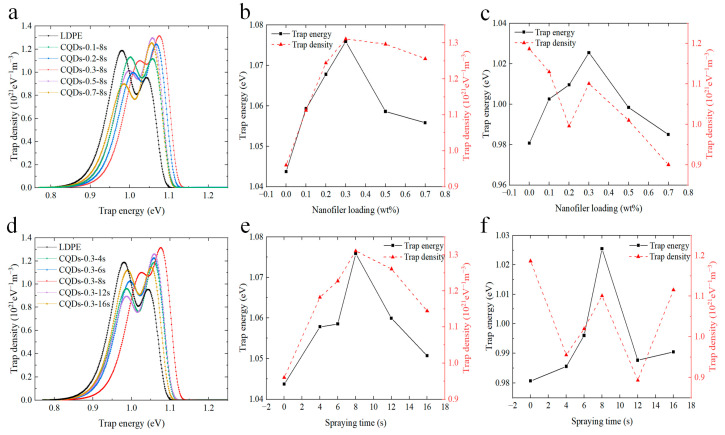
Trap level and trap density of different coated samples. (**a**) The surface coated with CQDs with different wt%; (**b**) extracted deep trap level and density of (**a**); (**c**) extracted shallow trap level and density of (**a**). (**d**) The surface coated with varying spraying durations; (**e**) extracted deep trap level and density of (**d**); (**f**) extracted shallow trap level and density of (**d**).

Figure 6 presents the trap-distribution characteristics of the composite coating. It is evident from Figure 6a,d that both the pristine LDPE and composite coating samples manifest a “bimodal” distribution in terms of trap energy levels. That is, two kinds of charge traps with shallower and deeper energy levels are generated in the coatings. Figure 6b,c describes the alterations in the distributions of deep and shallow traps with the concentration of CQDs. As can be seen, the energy level of shallow traps falls within the range of 0.98 to 1.03 eV, while the energy level of deep traps resides within the range of 1.04 to 1.08 eV. As the doping concentration of CQDs escalates from 0.1 wt% to 0.3 wt%, the energy levels of both deep and shallow traps elevate, accompanied by a gradual increase in the density of deep traps. Moreover, the density of shallow traps experiences a substantial reduction in comparison to that of pure LDPE, signifying a gradual increase in deep trap formation within the composite coating, while the occurrence of shallow traps is significantly diminished. The increase in both the energy level and density of deep traps impedes the process of detrapping, leading to a deceleration in the rate of surface potential decay [43,44]. With an incremental rise in the doping concentration of CQDs to 0.7 wt%, the energy level of both deep and shallow traps decreases, accompanied by a decrease in deep trap density. Consequently, this facilitates the detrapping and migration of trap charges, resulting in an upswing in the rate of surface potential decay (as shown in Figure 3a).

Figure 6e,f illustrates the trap distribution of coated samples with different spraying durations. The results indicate that the deep and shallow trap energy levels of coated samples exhibit higher values compared to LDPE. Furthermore, the increase in spraying time initially leads to an increase in the deep trap energy level and density, followed by a subsequent decline. Remarkably, the maximum deep trap energy level and density are achieved for the sample with a spraying time of 8 s. Simultaneously, the shallow trap energy level also exhibits a preliminary rise followed by a subsequent decline, although the correspondence between shallow trap density and spraying time is not particularly prominent. This suggests that spraying at the proper time helps to increase the trap energy level and deep trap density of the coated samples.

The deep traps introduced in the coated samples are caused by the different electronic energy band structures of the doped filler and the polymer matrix [45,46]. CQDs, as a semiconducting material, have a narrower energy band gap, which is capable of trapping free carriers through strong electrostatic attraction [47]. The electron trap depth is the energy difference between the LUMO levels of the pristine and doped polymers [48]. When the doping concentration is low, the large energy level difference leads to the introduction of deep traps in the band gap of LDPE due to the fact that the band gap of CQDs is about 3.2 eV while the band gap of LDPE is about 8 eV [49,50,51]. The charge injected from the electrode is captured by these deep traps, which reduces the charge injection and the carrier mobility and enhances the breakdown strength. When too many CQDs are introduced, the overlap of the interfacial region between the polymer and the filler reduces the energy level of the traps, which leads to a reduction in the breakdown strength.

## 4. Relation between Trap Properties and DC Breakdown

**Figure 7:**
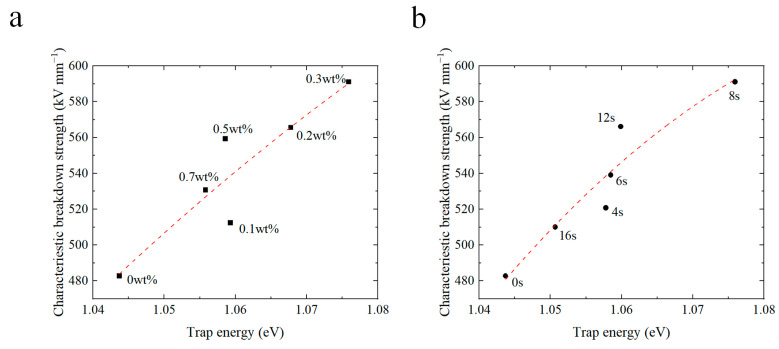
Relationship between the energy of deep traps and breakdown strength of coated samples doped with different concentrations of CQDs (**a**) and prepared with different spray durations (**b**).

**Figure 8:**
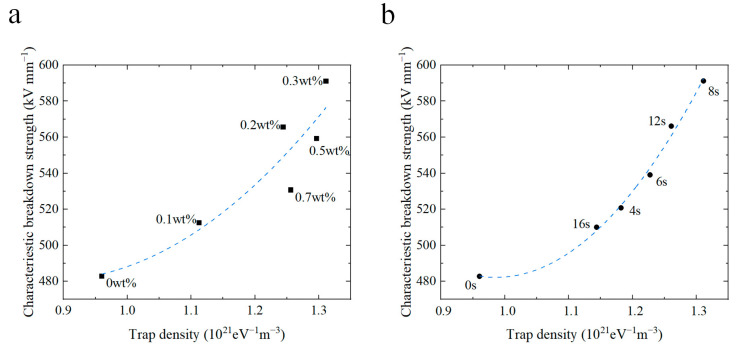
Relationship between the density of deep traps and breakdown strength of coated samples doped with different concentrations of CQDs (**a**) and prepared with different spray durations (**b**).

The introduction of a composite coating at the electrode–polymer interface brings forth a considerable amount of charge traps. In order to gain a deep insight into the impact of deep trap energy levels and densities on the breakdown strength, the correlation between the trap energy parameters and the DC breakdown strength was meticulously charted (as illustrated in Figure 7 and Figure 8). Figure 7 illustrates the interplay between the energy levels of deep traps and the breakdown strength. Notably, for coated samples with varying CQD concentrations and spraying durations, there is a conspicuous increase in the breakdown strength with the deep trap energy levels. Figure 8 demonstrates the impact of trap density on the DC breakdown strength. The breakdown strength of the coated samples increases with trap density. The conductivity of the coated samples follows a similar pattern, decreasing with increasing depth and density of the deep traps. 

The observed phenomenon can be attributed to the introduction of CQDs, which engender the formation of deep traps at the interface region between the nanofiller and the matrix. Consequently, charges injected into the electrode become entrapped within these deep traps within the coating, which generates a reversed electric field. As a result, the electric field in the vicinity of the electrode wanes, thereby inhibiting charge injection and mitigating electric field distortion. This process, in turn, diminishes the extent of field strength distortion and ultimately enhances the DC breakdown strength. Moreover, the increase in trap energy levels and density requires carriers to draw additional energy from the external electric field to surmount potential barriers [52]. As a result, the breakdown strength increases with the energy level and density of charge traps.

**Figure 9:**
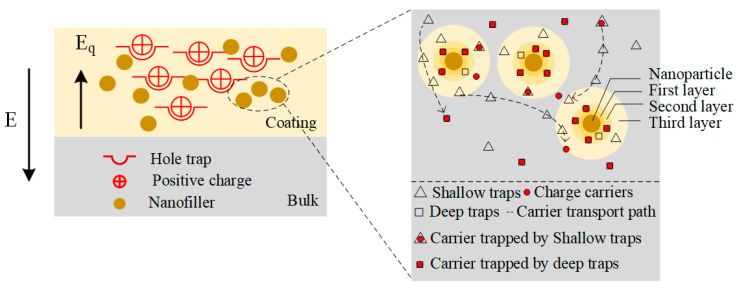
Mechanism schematic of insulation improvement.

**Figure 10:**
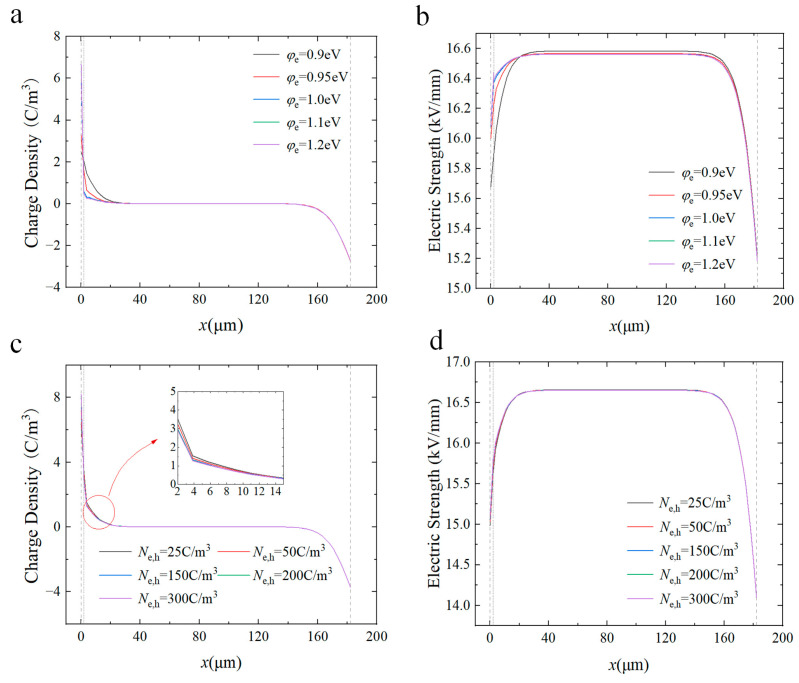
The influence of trap parameters on the distribution of space charge and electric field. Simulation results of space charge distribution (**a**) and electric field distribution (**b**) in coated samples for trap depths varying from 0.9 to 1.20 eV. Simulation of space charge distribution (**c**) and electric field distribution (**d**) in coated samples for trap density varying from 25 C/m^3^ to 300 C/m^3^.

A schematic diagram of the mechanism by which the interfacial composite coating modifies the insulating properties is given in Figure 9. The CQD nanoparticles in the coating sprayed at the surface of the film will form charge deep traps at the interface between the nanoparticles and the polymer matrix. According to the multi-core model proposed by Tanaka, the interface between the nanoparticles and the polymer matrix can be divided into a bonded layer (first layer), a bound layer (second layer), and a loose layer (third layer), and the deep traps are heavily distributed in the first and second layers, while the shallow traps are mainly distributed in the third layer [53,54]. Therefore, the introduction of interfacial composite coatings introduces deep traps between the electrode and polymer interfaces. At relatively low content of nanoparticles, the interfacial region between nanofiller and matrix is almost independent, so the charge injected from the electrodes is easily captured by the deep traps in the bonded and bound layers, which creates a reverse electric field (Eq) and inhibits the charge injection [55,56]. In addition, a large amount of charge is trapped during transport, leading to a decrease in carrier mobility, so that very little charge reaches the interior of the polymer, inhibiting space charge accumulation. As a result, deep traps lead to a concurrent decline in carrier concentration, subsequently diminishing the conductivity of the composite coating. And when the concentration of nanoparticles is high (above 0.3 wt%), the interfacial regions around neighboring particles overlap, leading to the expansion of the loose layer (transition region), which reduces the energy level and density of deep traps within the composite coating. And thus, carriers are easy to detrap from the traps, and the conductivity increases and the breakdown strength decreases. Figure 10 shows the simulation results of space charge distribution and electric field distribution at different trap energy parameters after the introduction of the interfacial composite coating. As can be seen, as the depth and density of the deep traps increase, more charges injected from the electrode are trapped and accumulated near the electrode, resulting in a lower charge migrating into the interior of the polymer medium and a lower degree of electric field distortion. For these reasons, the insulating performances, including breakdown strength and electrical conductivity, are effectively improved.

In addition, appropriate spraying durations can also enhance the insulating properties. We believe that it may be because the surface area of the film is not large and repeated spraying caused the coating buildup, which changed the distribution density of the CQDs. When the spraying time is short, the density of the CQDs increases, but the interaction zones around the neighboring particles are still almost independent, and the increase in the CQDs also results in the introduction of more deep traps; whereas, when the spraying time is too long, the coating buildup is severe, resulting in a considerable density of CQDs, the spacing between neighboring particles decreases, and even the interaction zones around neighboring particles overlap [57]. In conclusion, when over-spraying or spraying nanoparticles with a considerable coating density, the interfacial regions of nanoparticles overlap, thus the energy level and density of the deep traps within the composite coating are reduced. And carriers are easily detrapped from the traps, which generates a space charge accumulation inside the polymer, and a decrease in the breakdown strength occurs [58]. 

In essence, the introduction of an interfacial composite coating gives rise to a substantial abundance of deep traps situated between the electrode–polymer interface. These deep traps effectively intercept charge carriers, thereby engendering a reduction in their mobility. Simultaneously, the injected charges are captured by the deep traps inherent in the interface coating, prompting the emergence of homopolar charges in close proximity to the electrode. This phenomenon, in turn, reduces the electric field surrounding the electrode, thereby impeding charge injection. Consequently, the accumulation of space charges within the polymer medium is inhibited, and the distortion of the electric field is attenuated. Furthermore, the augmentation of trap energy levels necessitates that carriers acquire additional energy from the external electric field to surmount potential barriers. These collective factors synergistically contribute to the enhancement of the DC breakdown strength performance exhibited by the composite coating. 

## 5. Conclusions

In order to improve the electrical insulation properties of LDPE films, a composite coating doped with CQD particles was constructed between the electrode–polymer interface. The findings demonstrate that a judicious selection of filler doping concentration and spraying duration can effectively diminish the conductivity while increase the breakdown strength of LDPE films. A maximum increment of 22.4% was achieved for the breakdown strength. However, it is noteworthy that in cases where the filler doping concentration exceeds optimal levels or the spraying time extends beyond a certain threshold, the breakdown strength experiences a decline instead. The enhancement of insulation performance can mainly be attributed to the introduction of abundant deep traps at the surface of LDPE films. These traps impede charge injection, which suppresses the electric field distortion and elevates the breakdown strength to a superior level. The electrode–polymer interfacial composite coating provides a new idea to improve the insulation performance of polymer dielectrics.

## Data Availability

The data presented in this study are available on request from the corresponding author.
